# Innovative Hybrid Nanocomposites in 3D Printing for Functional Applications: A Review

**DOI:** 10.3390/molecules29215159

**Published:** 2024-10-31

**Authors:** Nguyen Thi Kim Tuyen, Dong Min Kim, Jung-Woo Lee, Jaehan Jung

**Affiliations:** 1 Department of Materials Science and Engineering, Hongik University, Sejong 30016, Republic of Korea; 2 Department of Materials Science and Engineering, Seoul National University of Science and Technology, Seoul 01811, Republic of Korea

**Keywords:** 3D printing, functional devices, hybrid nanocomposites

## Abstract

3D printing has garnered significant attention across academia and industry due to its capability to design and fabricate complex architectures. Applications such as those requiring intricate geometries or custom designs, including footwear, healthcare, energy storage, and electronics applications, greatly benefit from exploiting 3D printing processes. Despite the recent advancement of structural 3D printing, its use in functional devices remains limited, requiring the need for the development of functional materials suitable for 3D printing in device fabrication. In this review, we briefly introduce various 3D printing techniques, including material extrusion and vat polymerization, and highlight the recent advances in 3D printing for energy and biomedical devices. A summary of future perspectives in this area is also presented. By highlighting recent developments and addressing key challenges, this review aims to inspire future directions in the development of functional devices.

## 1. Introduction

Three-dimensional (3D) printing, also known as additive manufacturing, enables complete freedom in fabricating products across various sectors, such as the footwear [1], healthcare [2,3], automotive [4], aerospace [5], construction [6], energy storage [7,8], and electronics sectors [9]. This process supports the creation of complex geometries and architectures with varying thicknesses and at low costs, using a layer-by-layer deposition method without the need for templates [10]. Three-dimensional printing is an innovative technique for building objects ranging from the nanoscale to the microscale [11,12], with precise geometries directly from computer-aided-design models by accumulating materials in a layer-upon-layer manner [13]. A variety of 3D printing techniques have been developed, including vat polymerization (such as stereolithography (SLA), digital light processing (DLP), two-photon polymerization (TPP), and continuous liquid interface production (CLIP)) and material extrusion (e.g., fused deposition modeling (FDM) and direct ink writing (DIW)). Vat photopolymerization selectively cures photosensitive liquid resin to form 3D structures, offering high speed, precision, and smooth surfaces. Material extrusion is widely used owing to its simple mechanism and low-cost fabrication. In extrusion-based 3D printing, materials are extruded from a nozzle using mechanical force and then solidified to form 3D structures [14]. The build platform moves up and down as each layer is deposited. This technology has also been applied in various fields, including energy devices [15], biomaterials [16], and soft actuators [17].

Three-dimensional printing offers several significant advantages when applied to energy and electronic devices: (1) the ability to fabricate complex architectures with precise control over the shape and thickness, (2) high customization potential, and (3) cost-effectiveness, environmental friendliness, and ease of operation compared to conventional manufacturing process [18,19,20]. Consequently, 3D printing opens up new possibilities for the rapid fabrication of intricate 3D-structured energy storage and electronic devices with enhanced performance. In early studies, 3D printing technologies were primarily used to fabricate complex architectures that functioned mainly as scaffolds. Applications in fields like electrochemistry particularly benefited from the large surface area of electrodes made using 3D-printed scaffolds. However, additional post-processing steps, such as coating via CVD or electroplating, were often necessary. With advancements in organic–inorganic nanocomposites, functional devices like batteries and capacitors can now be directly 3D printed. Hybrid filaments or photocurable resins and inks are typically prepared by simply mixing active materials with thermoplastic or photocurable matrices.

In this review, we summarize the application potential of 3D printing in energy and electronic devices. The first section covers the fundamentals of various common 3D printing techniques, including vat polymerization (e.g., SLA, DLP, TPP, and CLIP) and material extrusion (e.g., FDM and DIW). The second section explores the applications of 3D printing in energy and electronic devices, with a focus on the design of cathodes, anodes, separators, and solid-state electrolytes, as well as its use in biomedical devices. In the final section, titled “Summary and Outlook”, we discuss the challenges and future prospects of the applications of 3D printing in energy and electronic devices, highlighting key directions for future research.

## 2. Three-Dimensional Printing Techniques

Vat polymerization (e.g., SLA, DLP, and TPP) and material extrusion (e.g., FDM and DIW) are widely utilized for fabricating functional devices. In material extrusion, desired materials are extruded through a nozzle to form 3D patterns layer by layer. In contrast, vat polymerization relies heavily on photocurable materials to build 3D structures.

### 2.1. Material Extrusion

Material extrusion is categorized into two main sub-types: thermoplastic material extrusion, commonly known as fused deposition modeling (FDM), and ink material deposition, also known as direct ink writing (DIW). Both FDM and DIW involve selectively depositing material through a nozzle or aperture. Unlike DIW, FDM can print freestanding structures without any additional modifications or ex situ curing. In recent years, both technologies have gained particular interest due to the emergence of multi-material printer configurations, paving the path for applications regarding battery production, supercapacitors, and electronics.

FDM, also known as fused filament fabrication or filament freeform fabrication, was developed by S. Scott Crump and patented in 1989. FDM operates by extruding thermoplastic filaments layer by layer through a heated nozzle to build a 3D structure [21]. Specifically, the nozzle moves in the X and Y directions to complete each layer, while the platform descends in the Z direction to begin a new layer. This process is repeated until the part is fully formed [22]. FDM allows for the rapid creation of 3D objects with minimal material waste. The thermoplastic materials used for FDM feed fibers are diverse, including acrylonitrile butadiene styrene (ABS), polylactic acid (PLA), poly-phenylsulfone (PPSF), polyamide (PA), polyethylene terephthalate (PET), polyethylene terephthalate glycol (PETG), and polycarbonate (PC).

DIW, a manufacturing technique involving the extrusion of ink through a nozzle to create freeform structures, was first reported by Cesarano and Calvert in 1997 [22,23]. DIW is conceptually similar to FDM, but instead of thermoplastic filaments, it utilizes viscous inks [24,25,26]. During the printing process, a nozzle deposits ink layer by layer to build objects with controlled architectures. The ink then solidifies to form 3D objects through liquid evaporation, gelation, or temperature/solvent-induced phase changes [27,28,29]. DIW is compatible with a wide variety of materials, including colloidal suspensions, polymers, hydrogels, waxes, metals, and ceramics [30,31,32]. Its simplicity, low cost, wide material selection, and ability to fabricate complex 3D structures at room temperature make DIW one of the most popular 3D printing techniques for energy and electronic device fabrication [33,34,35].

### 2.2. Vat Polymerization

One of the main challenges with material extrusion is the nozzle-dependent rough surface of the final product, which often necessitates an additional finishing step [36,37,38]. With respect to achieving smoother surfaces with higher resolution, vat photopolymerization offers an alternative approach [39]. This technique uses a vat of liquid photopolymer resin to build a 3D structure layer by layer. After each layer is cured by a light source, the process is repeated to form the next layer [40]. However, traditional fabrication tends to be slow. To address this, several advanced variants have been developed, including stereolithography (SLA), digital light processing (DLP), continuous liquid interface production (CLIP), and two-photon polymerization (TPP) [33].

Chuck Hull first introduced the term “stereolithography (SLA)” in 1984 when he applied for a patent for the process, which was granted in 1987 [41]. SLA works through the photopolymerization of liquid resin, caused by UV irradiation directed according to the desired geometry. The resin is typically composed of photoinitiators and photocurable molecules. Upon UV exposure, the photoinitiator, a light-reactive component, absorbs light and produces free radicals, which, in turn, trigger the polymerization of the resin [42,43,44]. Commonly used resin materials include acrylate, epoxy, urethane acrylate, and vinyl ether [45]. Acrylate-based resins, known for their fast curing speed and high reactivity [46], are especially suitable for SLA. These resins can be optimized for mechanical and thermal properties by adjusting the number of reactive groups [42] or by employing different oligomers, such as urethane-based acrylates [47]. However, acrylate resins tend to shrink during curing, potentially causing distortion. To mitigate this, they are often combined with methacrylates [48].

In contrast, epoxy-based resins use a cationic photopolymerization process rather than radical polymerization, which requires longer reaction times and is sensitive to moisture, though not to oxygen [49]. Epoxy exhibits lower shrinkage than acrylate [50]. Hybrid systems, combining acrylates and epoxies, have been developed to benefit from the strength of both, offering fast curing with minimal shrinkage, and they are now widely used in commercial SLA systems [51,52].

The SLA process starts by selectively solidifying a layer of resin with a laser to form a 3D pattern. After each layer is cured, the structure is submerged in the resin vat to a depth matching the layer thickness, allowing new layers to form on top of the existing structure. UV laser penetration exceeds the layer thickness, ensuring that each new layer adheres to the previous one through over-curing [53]. After fabrication, the 3D structure is placed in a developing solution to remove un-polymerized resin. A post-curing step with high-intensity UV radiation then fully polymerizes the structure, hardening it and reducing toxicity by eliminating residual monomers and oligomers. This post-curing process is especially useful for thick structures [54].

Since SLA uses a laser light source, it allows for high consistency and surface quality. The accuracy of SLA can be further enhanced by improving the focus of the laser spot [55]. SLA’s ability to print complex structures relatively quickly makes it a promising technique for fabricating energy and electronic devices [56].

Larry Hornbeck of Texas Instruments devised digital light processing (DLP) in 1987, a technique that shares many similarities with SLA. Unlike SLA, where a laser traces the desired pattern, DLP projects light across the entire resin tray at once, curing an entire layer simultaneously. The key to achieving the required shape for each layer lies in the illumination patterning, which is controlled by a digital micro-mirror device (DMD). It is located between the UV-emitting lamp’s optical path and the resin. The DMD consists of computer-controlled micro-mirrors mounted on a semiconductor chip. These tiny mirrors can tilt to either an “on” or “off” state: when tilted “on”, they reflect light to create a bright pixel, while the “off” state results in a dark pixel [14].

Because the DMD has a fixed number of mirrors, the image is scaled down as the printed object’s size increases, which can reduce the resolution of larger prints compared to smaller ones. Despite this limitation, DLP offers distinct advantages over laser-based SLA, such as lower costs and fast printing speeds, since it cures the entire surface of a layer at once [57,58].

Two-photon polymerization (TPP) is analogous to SLA but employs femtosecond laser to induce photopolymerization [59]. Excitation of photoinitiator molecules and material solidification can occur inside the diffraction limit because solidification in TPP occurs only when two photons are simultaneously absorbed. In detail, the polymerization process involves the interaction of two or more photons from a laser of a specific wavelength, which focuses on a designated point within a liquid photopolymer. After a predetermined volume of the material has been polymerized, the laser’s focal point is moved to create subsequent layers. The precision in controlling the size of the focal point and its positioning directly influences the final resolution and surface quality [60].

Two-photon absorption is achieved using a femtosecond laser, which is typically focused using standard microscope objectives. The size of the printed feature can be scaled by adjusting the numerical aperture of the focusing optics. Galvano-mirrors provide faster processing speeds compared to traditional translational stages [59]. TPP offers several advantages over conventional methods for the scalable fabrication of small-scale devices, enabling the production of arbitrary 3D micro/nanostructures from various materials, including polymers [61], hybrid materials, organically modified ceramics [62], and metals, all with resolutions beyond the sub-diffraction limit (down to 100 nm) [63,64].

The continuous fluid interface manufacturing (CLIP) technique is an advanced DLP-based technology developed by Carbon 3D Corp (Redwood, CA, USA) in 2015, serving as a new alternative to traditional layer-by-layer SLA [65]. CLIP shares similarities with DLP in that both utilize identical light sources and imaging systems, allowing the resins used in DLP to also be applicable in CLIP 3D printing [66]. Distinctively, CLIP utilizes an oxygen permeation membrane, which facilitates continuous printing by creating an oxygen-containing zone. This layer is located between the surface of the cured product and the image projection plane. The presence of the oxygen-permeable membrane enables the formation of a continuous liquid interface near the cured product, as oxygen inhibits the radical polymerization at the surface of the DLP window. Consequently, CLIP allows for faster fabrication compared to DLP, where UV curing, part movement, and resin renewal must occur sequentially [67].

## 3. Application of 3D Printing for Functional Devices

**Figure 1:**
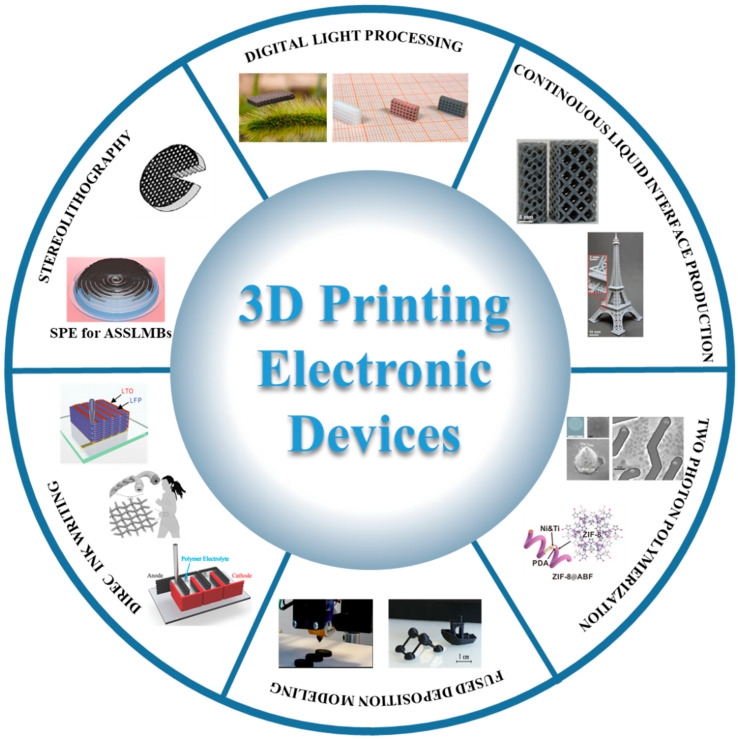
Examples of functional devices manufactured through diverse 3D printing techniques.

Three-dimensional printing technologies have been utilized in various fields, including energy [1], medicine delivery [2,3], and electronics [4,68,69,70], as illustrated in Figure 1. Recently, significant advancements have been achieved in the design and fabrication of complex architectures for functional devices through different 3D printing techniques, including fused deposition modeling (FDM), direct ink writing (DIW), two-photon polymerization (TPP), and stereolithography (SLA) [5,6,71].

### 3.1. Printing of Functional Materials via Material Extrusion

**Figure 2:**
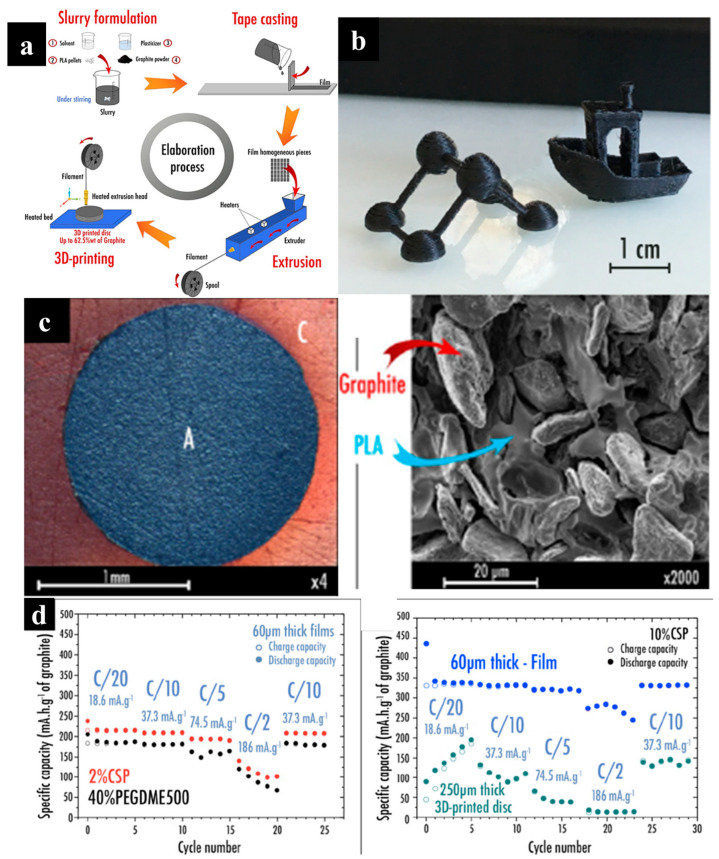
(**a**,**b**) Illustration of the synthetic process for fabricating fibers used in 3D printing of electrode disks, alongside examples of printed 3D objects made from 40% PEGDME filament. (**c**) Optical and SEM images of the 40% PEGDME500 homogeneous filament. (**d**) Electrochemical performance of printed electrodes fabricated using 40% PEGDME (includes the following configurations: 2% CSP (60 μm thick), 10% CSP (60 μm thick), and 10% CSP (250 μm thick)). Reproduced with permission from ref. [36]. Copyright @ 2018 American Chemical Society.

A variety of non-functional mechanical architectures have been fabricated using material extrusion techniques such as FDM and DIW. However, directly printing functional active materials for devices has been challenging, as it requires the development of hybrid filaments for FDM or specialized inks for DIW. In response, functional filaments and inks for electrodes, separators, and current correctors have been developed. Functional filament for FDM can be synthesized by simply mixing thermoplastic and functional materials such as conductor and functional particles. In a previous study, a 3D-printable graphite/PLA filament was developed specifically for use as the anode in lithium-ion batteries via an FDM process, as depicted in Figure 2a [36]. To prepare the anode filament for FDM, a composite mixture of graphite, polylactic acid (PLA), plasticizer, and solvent was deposited on substrates to form a film. The composite film was then sliced into 4 × 4 mm pieces and extruded into filament. Dichloromethane (DCM) was chosen as the solvent due to its rapid dissolution of PLA and fast evaporation after slurry tape casting. Several plasticizers, such as propylene carbonate (PC), poly(ethylene glycol) dimethyl ether (PEGDME), and acetyl tributyl citrate (ATBC), were incorporated into the graphite/PLA filament to improve printability. Among them, it is reported that the addition of 0 wt% PEGDME (M_w_ = 500) resulted in the most printable filament owing to the reduced stiffness and enhanced ductility. This study also examined the influence of conductive additives, such as carbon nanofibers (CNF) and carbon black Timcal Super-P (CSP), on the fiber’s conductivity. Conductive additives were mixed with graphite at weigh fractions of 1%, 2%, and 10%. The 10% CSP sample demonstrated the highest conductivity. Using the anode-functional filament, high-resolution 3D structures, including a semi-cubic lattice and a “3Dbenchy”, were successfully printed, as shown in Figure 2b. An optical microscope image of the homogenous filament containing 40 wt% PEGDME (M_w_ = 500) is shown Figure 2c. SEM observation revealed that the graphite particles were uniformly distributed throughout the PLA matrix.

Noticeably, the printed electrodes outperformed previously FDM-printed negative electrode disks in terms of reversible capacities, attaining a value of 200 mAh g^−1^ at a current density of 18.6 mA g^−1^ (C/20) and 140 mAh g^−1^ at 37.3 mA g^−1^ (C/10) after six cycles (Figure 2d). This work highlights the potential of improving the resolution of 3D printers and developing multi-nozzle configurations to print complex 3D electrode architectures, with designs optimized through detailed modeling studies.

**Figure 3:**
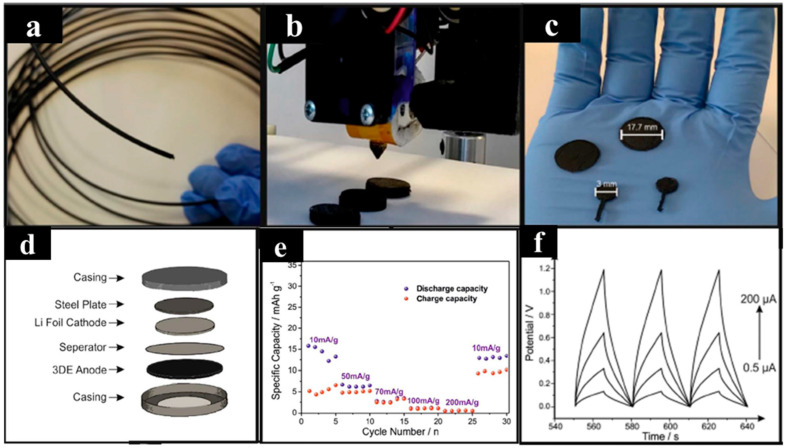
(**a**–**c**) Optical images of the graphene/PLA filament and 3D printing process. (**d**) Schematic representation of the coin cell structure. (**e**) Rate capability of the 3D-printed anode. (**f**) Galvanostatic charge–discharge profiles of the cells. Reproduced with permission from ref [72]. Copyright @2017 Springer Nature.

Additionally, 3D disk electrodes (3DEs) were fabricated utilizing a filament composed of 8 wt% graphene and 92 wt% of polylactic acid (PLA) via FDM 3D printing (Figure 3a–c) and applied as freestanding anodes for lithium-ion batteries and solid-state supercapacitors [72]. The 3D-printed lithium-ion freestanding anodes, with a thickness of 1 mm, were used to create coin cells without the need for current collectors (Figure 3d). The discharge capacities of the 3DE anodes were measured at different current densities, specifically at 15.8, 6.2, 2.6, 1.1, and 0.6 mAh g^−1^ of 3DE at 10, 50, 70, 100, and 200 mA g^−1^, respectively (Figure 3e). The relatively low discharge potential is attributed to the small amount of conductive material in the composite. Although the capacitance and specific capacity do not match those of advanced devices, they demonstrate the capability of fabricating low-cost, non-toxic 3D supercapacitor architectures (capacitance of 28 mF at 0.5 mA), as shown in Figure 3f.

In summary, the 3DE platform presents a foundation for next-generation 3D-printed energy architectures, offering several advantages: the fabrication of a freestanding electrochemical platform, elimination of the need for a metallic current collector for Li-ion anodes, the capability to fabricate a variety of geometrical shapes and sizes, the fact that there is no need for additional ex situ post-curing or modifications, and the potential to enhance electrochemical behavior by increasing oxygenated species on the surface of the 3D-printed material. These factors highlight the promise of 3D printing in energy storage applications.

Although functional filaments can be prepared through the straightforward physical mixing of active materials (e.g., graphene and graphite) with thermoplastics, the use of thermoplastics imposes limitations on the amount of active material that can be incorporated, hindering the development of truly functional filaments.

**Figure 4:**
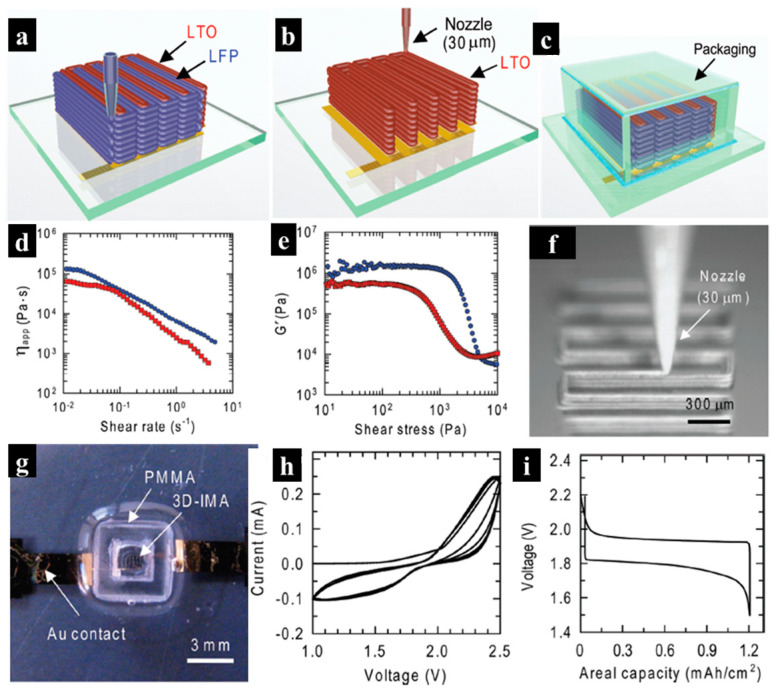
(**a**–**c**) Illustration of 3D interdigitated micro-battery (3D-IMA) architectures fabricated using Li_4_Ti_5_O_12_-anode and LiFePO_4_ (LFP)-cathode inks, extruded through 30 μm nozzles, followed by sintering and packaging. (**d**) Ink viscosity as a function of shear rate. (**e**) Storage modulus as a function of shear stress for each ink. (**f**) Optical image showing the deposition of LFP ink (60 wt%) through a 30 μ m nozzle to form a multilayer structure. (**g**) Optical image of 3D-IMA composed of LTO-LFP electrodes after packaging. (**h**) Cyclic voltammetry of the packaged 3D-IMA. (**i**) Charge and discharge curves of the packaged 3D-IMA. [Red and blue represent LTO and LFP inks, respectively]. Reproduced with permission from ref. [7]. Copyright @2018 John Wiley and Sons.

Compared to FDM, DIW offers greater flexibility in achieving functional inks for various applications, as a wide range of solution-processable organic–inorganic hybrid nanocomposites can be easily incorporated into printable inks [73]. Therefore, DIW has been extensively utilized to fabricate functional devices, as functional ink can be readily prepared by mixing active materials with a suitable solvent. The DIW technique was employed to fabricate a 3D-printed battery featuring an interdigitated architecture [7]. In detail, both a Li_4_Ti_5_O_12_ (LTO) anode and a LiFePO_4_ (LFP) cathode were printed onto a gold/glass substrate by depositing viscoelastic LTO and LFP inks through 30 μm cylindrical nozzles, as depicted in Figure 4a. The anode and cathode inks were synthesized by mixing LTO/LFP nanoparticles with cellulose-based viscosifiers in a solution comprising DI water, ethylene glycol (EG), and glycerol. The LTO mixture consisted of 27 wt% glycerol, 20–30 wt% EG, 9 wt% hydroxypropyl cellulose (HPC), 1 wt% hydroxyethyl cellulose (HEC), and DI water, while the LFP mixture contained 20 wt% glycerol, 20–30 wt% EG, 8 wt% HPC, 2 wt% HEC, and DI water. It should be noted that both inks show shear-thinning behavior, with viscosities ranging from 10^3^ to 10^4^ Pa · s at 1 s^−1^ (Figure 4d) and possess an adequate storage modulus (plateau modulus of ~10^6^ Pa and shear yield stress ranging from 10^2^ to 10^3^ Pa, Figure 4e) for 3D printing, thereby ensuring structural stability. Owing to their high viscosity, electrodes with aspect ratios ranging from 0.8 to 11 were fabricated through multilayer deposition of these inks (Figure 4f). After deposition, the electrodes were sintered at 600 °C in an inert atmosphere to remove organic additives. The addition of electrolyte (1M LiClO_4_ in a 1:1 volume ratio of ethylene carbonate to dimethyl carbonate), followed by passivation with PDMS gel and PMMA film, results in the final micro-batteries (Figure 4g). Figure 4h displays cyclic voltammetry characterization between 1.0 and 2.5 V at a scan rate of 5 mV s^−1^, revealing stable reduction and oxidation peaks at 1.3 V and 2.4 V, respectively. The 3D-printed micro-battery exhibited a capacity of 1.2 mAh cm^−2^, as shown in the galvanostatic charge and discharge curve at a rate of 0.5 C (Figure 4i). The printed 3D lithium-ion micro-battery demonstrated a high areal energy density of 9.7 J cm^−2^ at a power density of 2.7 mW cm^−2^, substantiating the capability of 3D printing to construct high-aspect structures within a compact area. The 3D-printed interdigitated micro-battery thus offers significant advantages in auto-powered micro-devices due to its high areal energy and power density.

**Figure 5:**
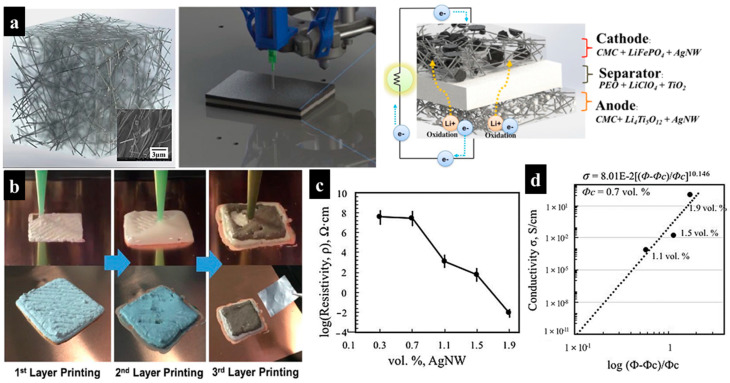
(**a**) Schematic representation of the AgNW/cellulose composite and 3D printing process for battery components. (**b**) Digital images showing several stages of the 3D printing process. (**c**,**d**) Resistivity plot showing the electrical properties of the 3D conductor. Reproduced with permission from ref. [74]. Copyright @2017 Springer Nature.

Park et al. fabricated a lithium-ion battery (LIB) with integrally printed electrodes and an electrolyte through DIW, as illustrated in Figure 5a [74]. In this study, high-aspect-ratio silver nanowires (AgNWs) were introduced, since they render lower percolation thresholds and offer high conductivity, reaching up to 10^3^ S/cm. Sodium carboxyme thyl cellulose (CMC) was selected as the matrix material due to its ability to thicken viscosity and exhibit thixotropic rheology. Cathode and anode inks were prepared by mixing lithium iron phosphate (LFP) and lithium titanate (LTO) with DI water, CMC, and AgNWs, respectively. The electrode pastes were made with a high concentration of solid particles (40 wt% active materials, CMC, and AgNW) to minimize volume shrinkage after drying. The electrolyte ink comprised acetonitrile, TiO_2_, lithium perchlorate (LiClO_4_), and polyethylene oxide (PEO). A full battery was fabricated by sequentially printing the anode, electrolyte, and cathode on a Cu foil current collector, followed by the application of Al foil to the cathode layer and a moisture removal procedure (Figure 5b).

It is reported that the electrical conductivity of this composite increased significantly when the AgNW ratio exceeded 0.7 vol%. The resistivity of the AgNW/CMC composite was 2.57 × 10^7^ Ω cm and 8.38 × 10^−3^ Ω cm at 0.7 vol% and 1.9 vol% of AgNW, respectively (Figure 5c,d). In comparison, the electrical conductivities of the pristine LFP (~10^−9^ S cm^−1^) and LTO (~10^−13^ S cm^−1^) electrodes were several orders of magnitude lower than those of the AgNW incorporated cathodes (4.21 × 10^−3^ S cm^−1^) and anodes (1.64 × 10^−4^ S cm^−1^). This research paves the way for 3D-printable conductive pastes to be used in next-generation additive manufacturing processes to make printed electronics.

**Figure 6:**
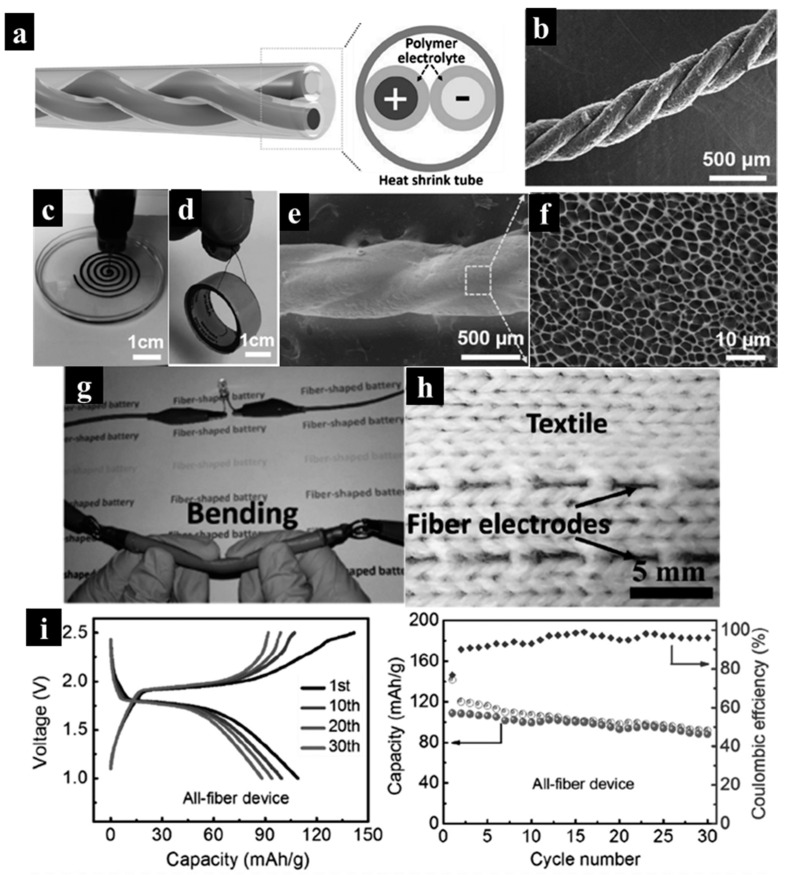
(**a**) Schematic of the all-fiber LIB device. (**b**) SEM image of yarn composed of three LFP fibers. (**c**,**d**) Optical image of a wet fiber during the printing process and a fiber bearing a burden. (**e**,**f**) SEM image displaying a polymer layer coated on the yarn fibers. (**g**) Demonstration of the all-fiber device powering an LED while in a bent state. (**h**) A photo image of the integration of fiber electrodes into textile fabrics. (**i**) Charge/discharge profiles and cycling stability of the all-fiber LIB. Reproduced with permission from ref. [75]. Copyright @2017 John Wiley and Sons.

Wang et al. utilized a DIW approach to examine 3D-printed all-fiber quasi-solid-state LIBs for wearable energy storage [75]. In this study, cathode and anode inks for 3D printing were prepared by mixing lithium iron phosphate (LFP) particles and lithium titanium oxide (LTO), poly vinylidene fluoride (PVDF), and carbon nanotubes (CNTs) in n-methyl-2-pyrrolidone (NMP) with a mass ratio of LFP/LTO:CNT:PVDF set at 6:3:10. A coating of poly (vinylidene fluoride-co-hexafluoropropylene) (PVDF-co-HFP) was applied to the surfaces of both LFP and LTO fibers to facilitate the absorption and retention of the liquid electrolyte (LiPF_6_-EC/DEC). The coated LFP and LTO fibers were then twisted together and encased in a tube, resulting in a single integrated fiber, as shown in Figure 6a,b. Specifically, each cathode/anode fiber was extruded into an ethanol solution from the 3D printing syringe in Figure 6c. The viscosity of the inks ranged from 10^4^ to 10^5^ Pa · s at a shear rate of 10^−2^ s^−1^ for cathode and anode inks. The extruded fiber measured 23 cm in length and 200 μm in diameter, exhibiting sufficient robustness to support a heavy ring (Figure 6d), which highlighted its inherent flexibility and mechanical strength. SEM studies (Figure 6f) revealed that the PVDF-co-HFP-coated yarn had a thickness of 8–16 μm and a porous surface, enabling localized absorption and storage of the liquid electrolyte. Thanks to the fibril nature of the batteries, they could power a light-emitting diode without degradation, even under a bending condition (Figure 6g). It was not surprising that weaved fabrics were achievable due to the fibers’ exceptional flexibility and mechanical strength (Figure 6h). An all-fiber LIB with high specific capacity (110 mA h g^−1^ at 50 mA g^−1^) and remarkable flexibility was achieved by twisting the printed LFP and LTO fibers with a quasi-solid-state gel electrolyte into a single yarn, as shown in Figure 6i. This advanced 3D printing technique enables rapid and efficient production of a low-cost all-fiber flexible design, presenting a promising solution for wearable electronics.

**Figure 7:**
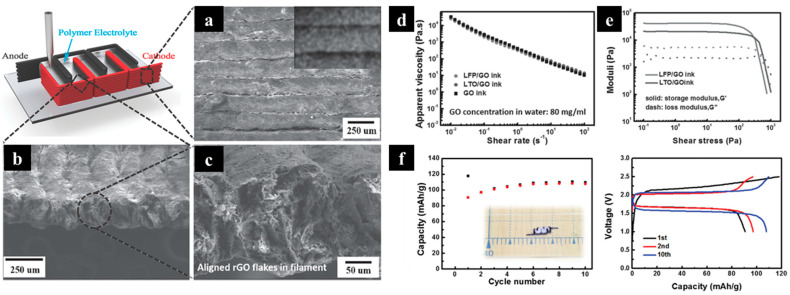
(**a**–**c**) Schematic of the 3D-printed interdigitated electrodes and their SEM images. (**d**) Apparent viscosity as a function of shear rate for pure GO, GO/LFP, and GO/LTO inks. (**e**) Storage modulus and loss modulus as a function of shear stress for GO/LFP and GO/LTO inks. (**f**) Cycling stability and charge/discharge profiles of the 3D-printed full cell, consisting of LFP/rGO, LTO/rGO, and polymer. Reproduced with permission from ref. [34]. Copyright @2016 John Wiley and Sons.

Fu and co-workers fabricated lithium-ion batteries with 3D-printed interdigitated electrodes and a solid-state electrolyte via DIW, as shown in Figure 7 [34]. The 3D printing of all-component batteries (electrodes and electrolytes) was reported by adopting adequate solution rheology of the electrolyte. The electrolyte ink composite was composed of PVDF-co-HFP and Al_2_O_3_ nanoparticles. The alumina nanoparticles were included to enhance electrolyte uptake and the liquid electrolyte storage sustainability in the polymer composite. For cathode and anode inks, LFP and LTO were selected and mixed with aqueous graphene oxdie (GO). GO, with its high specific surface area, also contributed to the ink’s viscosity and modified its rheological properties while improving the electrical conductivity of the electrodes after thermal annealing. Additionally, the use of water as a green solvent made this ink system feasible for environmentally friendly and low-cost processing.

The 3D-printed full cell was prepared by printing interdigitated electrodes with dimensions of 7 mm × 3 mm. The electrolyte composite (PVDF-co-HFP and Al_2_O_3_ nanoparticles) was then printed into the gaps between the interdigitated electrodes and soaked in a liquid electrolyte. A surface view of the LFP/rGO revealed a uniform distribution of LFP nanoparticles within the rGO matrix (Figure 7a). The inset SEM image in Figure 7b shows the tightly packed filaments, which promoted the superior electrical conductivity and mechanical integrity of the electrode. After the thermal annealing process, voids formed due to the release of gaseous products and the restacking of rGO flakes (Figure 7c).

High viscosity and shear-thinning behavior were observed in the GO-based electrode inks (Figure 7d). The results regarding the loss modulus (G″) and storage modulus (G′) of LFP/GO and LTO/GO inks (Figure 7e) showed that with an increase in shear stress, the loss modulus surpasses the storage modulus, indicating viscous deformation as the dominant behavior. The electrochemical performance of the full cell is displayed in Figure 7f, where the 3D-printed LFP/LTO demonstrates stable cycling performance, with specific capacities of approximately 160 mAhg^−1^ and 170 mAg^−1^, respectively. The full cell delivered initial charge and discharge capacities of 117 and 91 mAh g^−1^, with good cycling stability. This liquid deposition modeling, however, required post-treatment, such as freeze-drying or thermal annealing, to remove the solvent and solidify the 3D structure.

### 3.2. Printing of Functional Materials via Vat Polymerization

**Figure 8:**
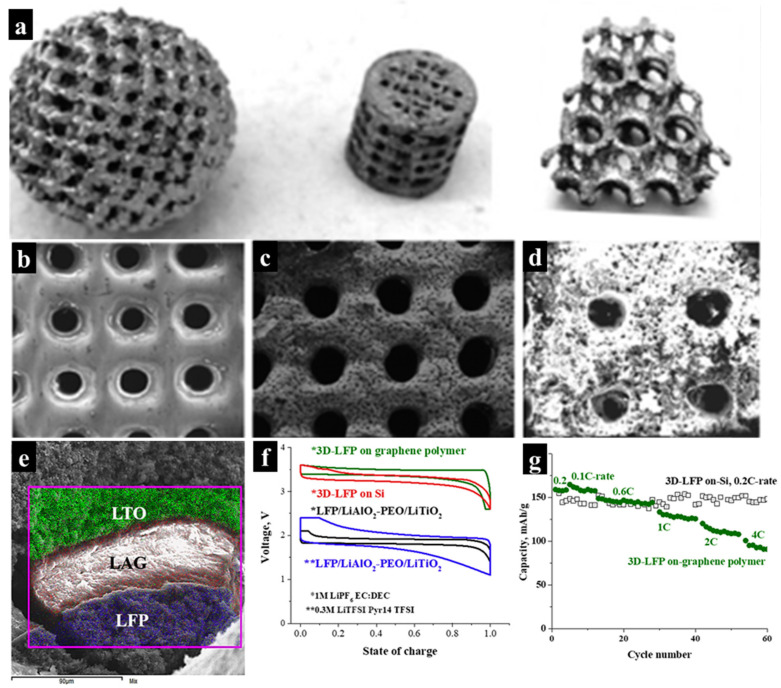
(**a**,**b**) Three-dimensionally printed perforated polymer substrates and the three-dimensional structure coated with (**c**) nickel, (**c**) a Ni-LFP layer, and (**d**) Ni-LFP-LAO:PEO layers. (**e**) Cross-sectional EDS mapping of tri-layer battery structures deposited on BLACKMAGIC3D. (**e**) Graphene 3D lab polymer substrate of LFP-LAGP: PEI-LTO layers. (**f**) Charge/discharge voltage profiles and (**g**) cycle performance of printed batteries. Reproduced with permission from ref. [76]. Copyright @2018 Elsevier.

Vat polymerization techniques, such as SLA, DLP, and TPP, offer higher resolution compared to material extrusion methods. Their use in functional devices has primarily focused on fabricating intricate morphologies for sacrificial scaffolds. It is well known that perforated structures are favorable for enhancing electrochemical reactions. In this regard, vat polymerization (SLA, DLP, and TPP) has been employed to fabricate complex structures, including both scaffolds and functional devices such as anodes or cathodes. For example, porous spherical, cylindrical, and cubic polymer substrates for high-energy and high-power thin-film 3D micro-batteries were fabricated using SLA technology in [76]. A simple and cost-effective electrophoretic deposition (EPD) method (Figure 8a–d) was used to create 3D-lithiated cathodes and anodes on the 3D-printed scaffolds. The tri-layered devices, as illustrated in Figure 8e, consist of a LiFePO_4_ (LFP) cathode, a LiAlO_2_-PEO or Li_1+x_Al_y_Ge_2−y_(PO_4_)_3_-PEI membrane, and a LiTiO_2_ (LTO)-based anode. Various binders (e.g., polyvinylidene fluoride (PVDF), poly methyl methacrylate (PMMA), lithium polyacrylate (LiPAA), and polyvinylpyrrolidone (PVP)) can be utilized within this system. The Li_1+x_Al_y_Ge_2−y_(PO_4_)_3_-PEI (LAGP) membrane was deposited using the AC-EPD method. The resulting cell demonstrated an aerial capacity of 400–500 mAh cm^−2^ at C-rates ranging from 0.1 to 4 (Figure 8f,g). It was reported that the areal energy density of the 3D micro-batteries on the silicon chip was at least one order of magnitude higher than that of commercial planar thin-film batteries owing to their increased surface area.

**Figure 9:**
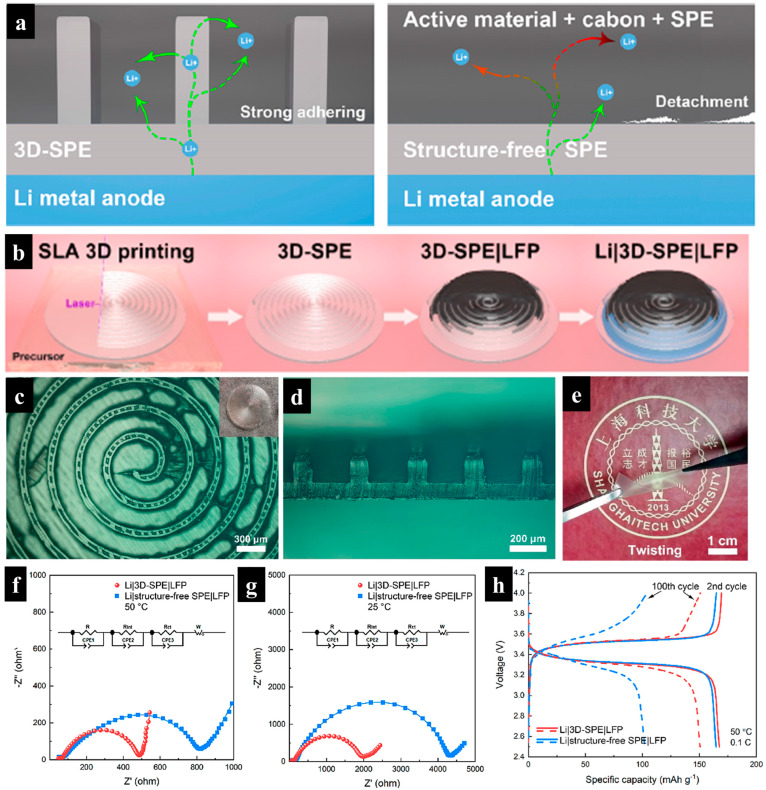
(**a**) Schematic illustration of all-solid-state Li metal battery (ASSLMB) comparing a 3D-structured solid polymer electrolyte (SPE) created via SLA 3D printing with structure-free SPE. (**b**) Schematic depiction of the preparation process for ASSLMBs utilizing 3D-SPE. (**c**) Top view and (**d**) cross-sectional image of the 3D-SPE. (**e**) Optical microscope image of flexibility SPE. (**f**,**g**) Nyquist plots of the half cells at 25 °C and 50 °C. (**h**) Cycling performances of Li|3D-SPE|LFP and Li|structure-free SPE|LFP cells at 0.1 C at 50 °C. Reproduced with permission from ref. [77]. Copyright @2020 American Chemical Society.

For all-solid-state lithium metal batteries (i.e., Li/3D-SPE/LFP cells), solid polymer electrolyte (SPE) was fabricated via SLA 3D printing [77]. This approach improves interfacial adhesion between electrolyte and electrode, increases specific area, and allows for larger active material loading by forming SPE with an Archimedean spiral structure (Figure 9a,b). In this work, poly (ethylene oxide) (PEO) polymer was utilized as the SPE due to its high ionic conductivity and photocurability. The resin consisted of poly (ethylene glycol) diacrylate (PEGDA), 1 wt% phenyl bis(2,4,6-trimethylbenzoyl)phosphine oxide, succinonitrile (SCN), and lithium bis(trifluoromethanesulfonyl)imide (LiTFSI) in a weight ratio of 1.5:1:1.4.

This mixture was photocured using a 355 nm wavelength laser to print the 3D Archimedean spiral PEO-based SPE. The resulting 3D-SPE featured a spiral pattern with a width of 100 μm, a height of 150 μm, and spacing of 200 μm on a planar substrate with a thickness of 100 μm. The solid-state Li/3D-SPE/LFP cells (Figure 9c–e) were fabricated and compared with cells utilizing structure-free SPE. The cells with spiral SPE showed a lower total impedance than those with structure-free SPE, attributed to the increased specific area between the cathode and electrolyte at both room temperature and 50 °C. These solid-state Li/spiral patterned SPE/LFP cells exhibited a high mass loading of 5 mg cm^−2^, a specific capacity of 166 mAh g^−1^, and a superior capacity retention of 77% after 250 cycles at 50 °C (Figure 9f–h). It should be noted that the spiral SPE structure formed by SLA printing improves performance by shortening the Li-ion transport channel from the electrolyte to the electrode and reinforcing interfacial adhesion during battery cycling. SLA offers a promising pathway for high-performance, scalable, all-solid-state lithium metal batteries in the future of energy storage.

**Figure 10:**
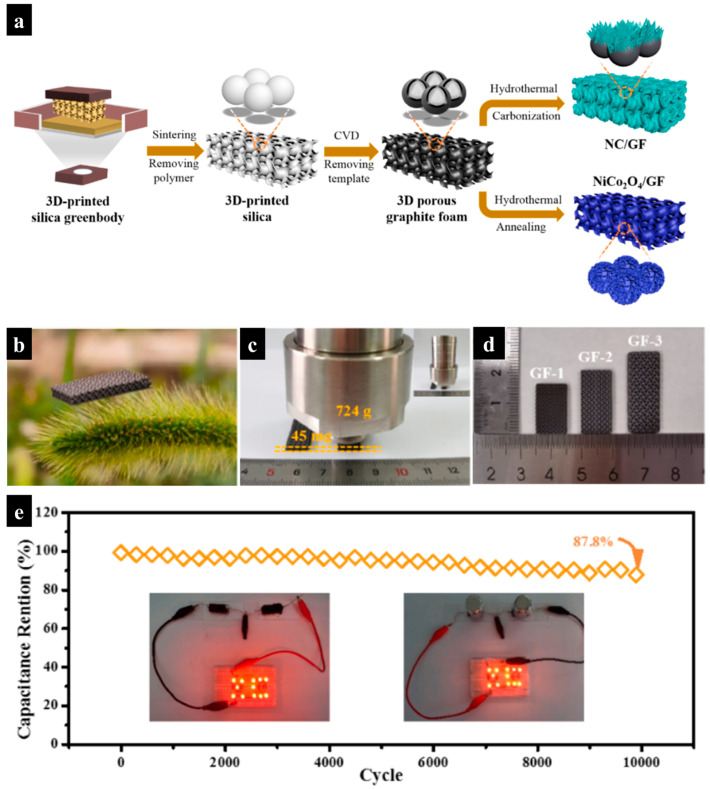
(**a**) Illustration of synthetic strategy for 3D porous graphite foam (GF)-based electrodes for supercapacitors. (**b**–**d**) Demonstration of the lightweight property and high stiffness exhibited by the 3D GF. (**e**) Cycling performance of the quasi-solid-state supercapacitor. Inset: LEDs powered by two quasi-solid-state supercapacitors in their initial state and under compression. Reproduced with permission from ref. [78]. Copyright @2021 Elsevier.

Recently, Du et al. developed a gyroidal hierarchical porous 3D graphite foam (GF) through a combination of digital light processing (DLP) and chemical vapor deposition (CVD), a method that leverages the advantages of high surface area in hierarchical structures for energy applications [78]. Figure 10a shows an illustration of the synthetic route for the 3D hierarchical porous GF-based electrodes. To prepare porous 3D graphite, a silica sacrificial template was first printed using a DLP printer, followed by the growth of graphite on a silica template through CVD. The silica template was then removed via hydrofluoric acid etching, leaving behind a nanoporous GF with a hollow feature.

The silica resin for DLP printing was prepared by mixing silica powder, Variquat CC 42 NS, 1,6-hexanediol diacrylate (HDDA), trimethylolpropane ethoxylate triacrylate (E-TMPTA), and diphenyl (2,4,6 trimethyl benzoyl)phosphine oxide (TPO). The resulting GF is incredibly light, as demonstrated by its ability to be supported by dogtail grass without bending the delicate hairs (Figure 10b). It also shows exceptional stiffness, withstanding a load 16,000 times its own weight without deforming (Figure 10c,d).

For capacitor applications, N-doped carbon nanosheet/GF and NiCo_2_O_4_/GF electrodes were prepared via hydrothermal processes. The assembled NiCo_2_O_4_/GF//NC/GF device showed a remarkable volumetric capacitance of 0.81 F/cm^3^ at a high current density of 75 mA/cm^3^. It retained 87.8% of its initial capacitance after 10,000 cycles, demonstrating excellent cycle stability (Figure 10e). The device was also able to power light-emitting diodes (LEDs) consistently, even under heavy loading.

This approach to 3D printing opens the door for the development of customizable, high-performance energy storage devices, demonstrating the potential of combining DLP and CVD techniques to create advanced, hierarchically structured materials for future energy applications.

**Figure 11:**
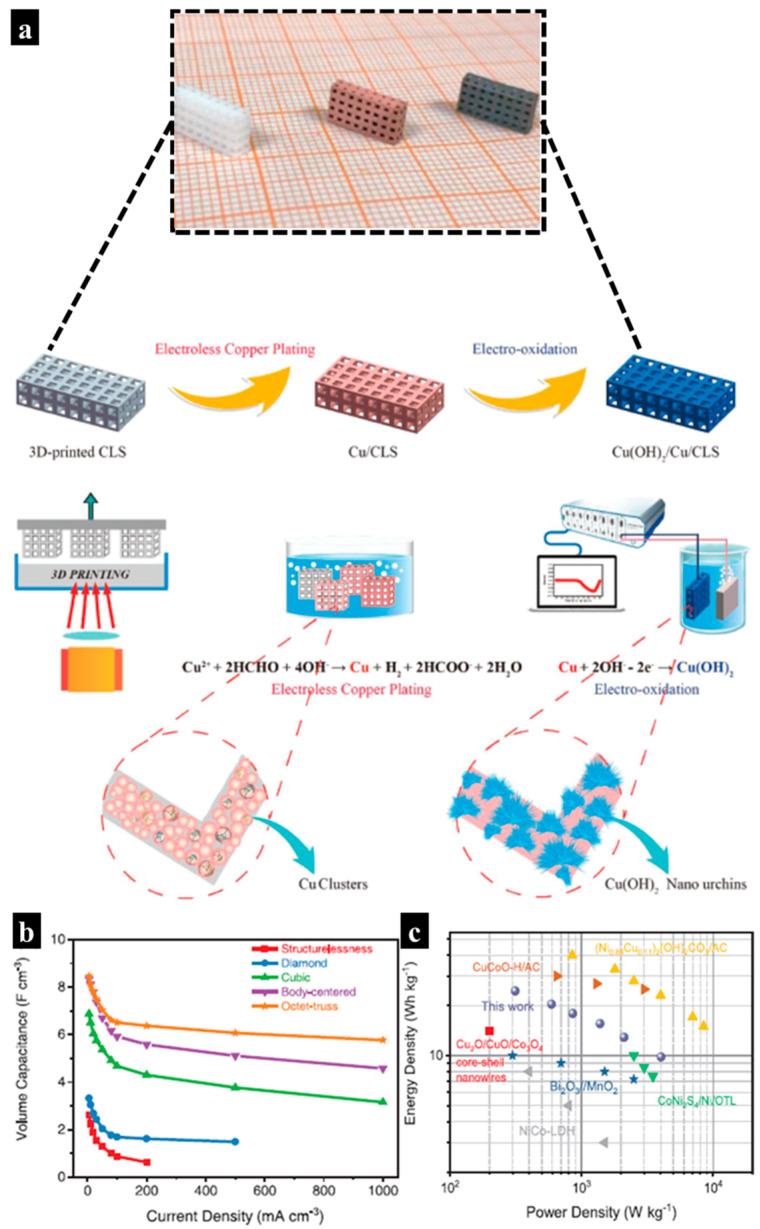
(**a**) Photograph showing the 3D-printed CLS, Cu/CLS, and Cu(OH)_2_/Cu/CLS, along with a schematic illustration of the synthetic procedure for Cu(OH)_2_/Cu/CLS. (**b**) Plots of volumetric capacitance versus current density for Cu(OH)_2_/Cu/CLS electrodes with different lattice structures. (**c**) Ragone plot of the symmetric Cu(OH)_2_/Cu/CLS supercapacitor. Reproduced with permission from ref. [79]. Copyright @2019 John Wiley and Sons.

By bridging 3D printing technology with a simple electroless plating and electro-oxidation process, a new path for constructing 3D structural supercapacitors based on urchin-like Cu(OH)_2_ lattice electrodes was reported by Chang and co-workers [79]. The process utilized DLP to create a periodic lattice structure using porcelite ceramic resin, which was subsequently immersed in a copper solution bath for electroless plating to deposit a layer of copper on the surface. Finally, the electro-oxidation process yielded a Cu(OH)_2_ nanourchins/Cu/ceramic lattice substrate (Cu(OH)_2_/Cu/CLS) (Figure 11a).

As shown in Figure 11b, the 3D-printed Cu(OH)_2_/Cu/CLS octet-truss electrodes demonstrated remarkable volumetric capacitances, delivering 8.46 F cm^−3^ at 5 mA cm^−3^ and 5.76 F cm^−3^ at 1 A cm^−3^, while maintaining nearly 84.3% of the discharging capacitance after 5000 cycles. The symmetric cell also showed a high capacitance of 6.28 F cm^−3^ at 5 mA cm^−3^, with excellent rate performance, retaining 60% of the capacitance at 200 mA cm^−3^. Additionally, it demonstrated long-term stability, retaining 70.2% of its capacitance over 5000 cycles.

The performance metrics of the fabricated symmetric supercapacitor were impressive, exhibiting both high energy and power densities of 24.4 Wh kg^−1^ and 4039.2 W kg^−1^, respectively (Figure 11c). These results highlight the ability to tune the volumetric capacity and mechanical strength of 3D lattice electrodes through simple 3D spatial framework designs. This technique offers significant potential for creating customizable power sources.

**Figure 12:**
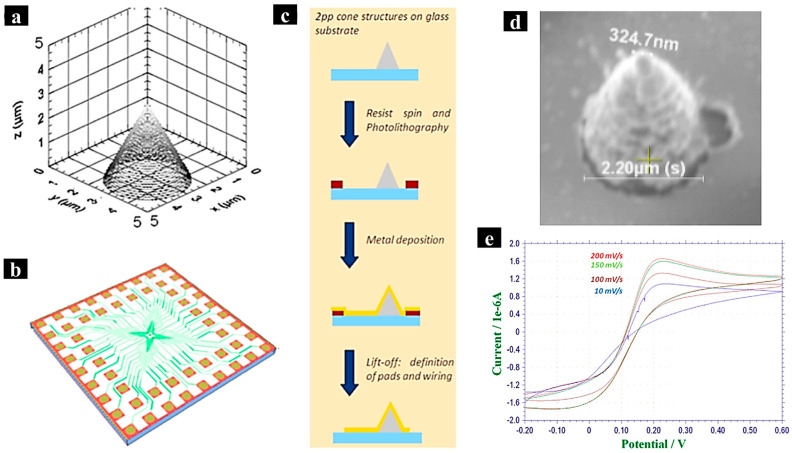
(**a**–**c**) A schematic representation of the single cone electrode, a flowchart of the process for integrated microfabrication, and the mask design. (**d**) An SEM image of the fabricated cone structure using IP-L photopolymer after metallization, achieved by focusing a femtosecond laser (6 mW power) onto I-PL. (**e**) Cyclic voltammograms of a single electrode in 10 mM ferri/ferrocyanide, with Ag/AgCl as the reference electrode and a Pt wire as the counter electrode. Reproduced with permission from ref. [80]. Copyright @2012 Elsevier.

Al-Abaddi et al. fabricated 3D cone structures as electrodes using two-photon polymerization, with interconnections created via UV photolithography [80]. A photoinitiator, Irgacure 369, and a commercially available negative-tone photopolymer, I-PL (from Nanoscribe GmbH), were used in this study (Figure 12a–c). A femtosecond laser beam with a wavelength of 780 nm and a pulse duration of 150 fs was focused through a high numerical aperture objective onto a droplet of the photopolymer, which was placed on a 30 mm thick glass substrate. Because IP-L is a negative resist, rhe unexposed area was washed away during the development process, leaving behind the polymerized structures. Hexamethyldisilazane (HMDS) was used to increase the adhesion between the glass substrate and the photoresist. The interconnections and pads were defined using AZ5214 resist, exposed through a mask, followed by metal deposition (10 nm titanium layer and 100 nm gold layers). A lift-off procedure resulted in fully fabricated 3D cone electrodes, as depicted in Figure 12d.

Electrochemical performance was characterized through voltammograms, which displayed a proportionate relationship between peak current and potential sweep rate, with no significant potential shift (Figure 12e). This indicates that the electrodes exhibit quasi-reversible electrochemical behavior, with good electrical connections and electronic characteristics achieved through the metallization process.

The two-photon polymerization technique offers a cost-effective method for fabricating micro/nanostructures and is expected to become a promising platform for the high-resolution 3D printing of functional structures. This innovative approach holds great potential for future applications in micro–nano production.

**Figure 13:**
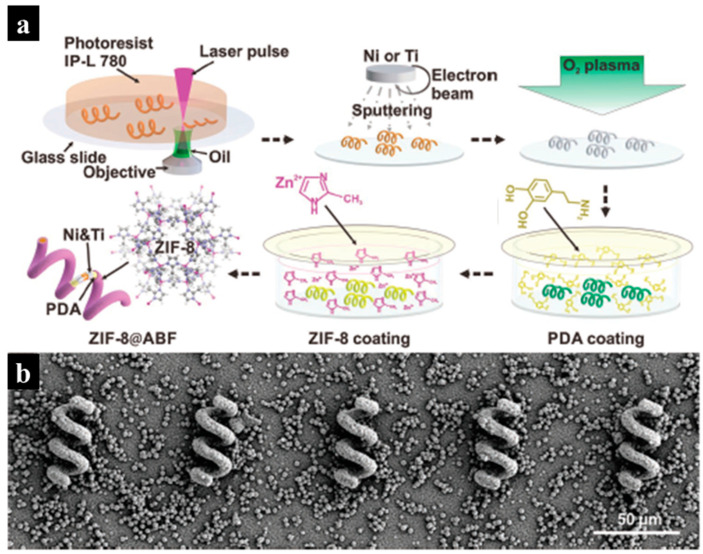
(**a**) An illustration of the fabrication process for the ZIF-8@ABF microrobots. (**b**) An SEM image of the ZIF-8@ABF microbots. Reproduced with permission from ref. [81]. Copyright @2019 John Wiley and Sons.

Wang et al. successfully developed magnetic helical microstructures, known as an artificial bacterial flagella (ABF), which were coated with zinc-based metal−organic frameworks (MOFs), using the two-photon polymerization (TPP) technique [81]. These structures, coated specifically with a zeolitic imidazole framework-8 (ZIF-8), serve as advanced microbots for targeted drug delivery applications. In this study, two-photon polymerization was employed to create a helical framework capable of supporting the growth of magnetic metals and MOF, as shown in Figure 13a. After printing the helical structure, nickel and titanium were deposited on the surface using an e-beam evaporator, followed by the introduction of polydopamine (PDA) on the ABF surface. ZIF-8 coatings finally yielded magnetic and pH-responsive ZIF-8@ABF microrobots, as shown in the SEM image in Figure 13b.

These microbots can navigate complicated microfluidic channel networks under the control of the magnetic field and serve as pH-responsive targeted drug delivery platforms, carrying and releasing medications. Beyond existing applications, this innovative method of manufacturing integrated multifunctional systems will offer new pathways in soft microrobotics.

## 4. Summary and Outlook

In this review, we first presented an overview of 3D printing techniques, including material extrusion and vat polymerization, and their applications in energy and electronic devices. Three-dimensional printing technologies have drawn significant attention across industries and academia due to their potential applications, including applications in the energy [7], medical modeling [82,83], and electronics sectors [84]. Recent advancements in the design and fabrication of complex architectures through 3D printing methods, including fused deposition modeling (FDM), selective laser sintering (SLS), and stereolithography (SLA) methods, have been noteworthy [85,86]. Despite these developments, the fabrication of microscale functional devices via 3D printing remains a challenge. There is a growing need for the development of hybrid functional composites for 3D printing to address this gap. Although several studies have reported the fabrication of functional devices through 3D printing [34,84], micro-batteries prepared via material extrusion techniques like DIW and FDM suffer from poor resolution and require post-curing steps (e.g., heat treatment). Even though DIW offers better resolution than FDM, it still greatly depends on the rheology of the ink materials. Vat polymerization techniques such as SLA are advantageous among the various 3D printing strategies, as they can offer a hundred-nanometer resolution at a low cost and without the need for post-curing [87,88]. However, the preparation of photocurable resin with functional materials is greatly required for use in these types of 3D printing.

Clearly, research on hybrid organic–inorganic nanocomposites for 3D printing techniques, including functional filaments, inks, and photocurable hybrid resins, needs to be advanced. So far, functional filaments for FDM have been created by simply mixing active materials with thermoplastics. However, surface modification of these active materials may enable more comprehensive device fabrication. In DIW, hybrid nanocomposites can be incorporated into functional inks to control rheological properties, thereby improving printing quality and reducing the need for post-processing treatments. Although functional inks, such as photoactive quantum dot ink for 2D pattering in LED applications, have been developed, their use in 3D fabrication remains challenging. Consequently, there is a pressing need to develop photocurable hybrid organic–inorganic resins that do not hinder interaction with UV light for vat polymerization. Moreover, resolution improvement will also remain a promising area of exploration for electronic devices derived from 3D printing.

## Data Availability

No new data were created or analyzed in this study. Data sharing is not applicable to this article.
